# Efficacy and safety data on pretomanid for drug-resistant TB

**DOI:** 10.5588/ijtldopen.24.0360

**Published:** 2025-02-01

**Authors:** H.T.T. Thuy, C. Padmapriyadarsini, C. Chuchottaworn, S. Foraida, S. Hadigal, A.R. Birajdar

**Affiliations:** ^1^ National TB Control Programme of Vietnam, National Lung Hospital, Hanoi, Vietnam;; ^2^ National Institute for Research in Tuberculosis, Indian Council of Medical Research, Chennai, India;; ^3^ Department of Medical Services, Central Chest Institute of Thailand, Nonthaburi, Thailand;; ^4^ TB Alliance, New York, NY, USA;; ^5^ Department of Medical Affairs, Viatris, India.

**Keywords:** tuberculosis, extensively drug-resistant tuberculosis, pre-extensively drug-resistant tuberculosis, multidrug-resistant tuberculosis, adverse events

## Abstract

**OBJECTIVE:**

To summarise the efficacy and safety of pretomanid (Pa) based regimens in patients with drug-resistant TB (DR-TB).

**METHODS:**

We included clinical trials, operational research and observational studies reporting the efficacy and safety of Pa-based regimens in DR-TB. The duration of the treatment was at least 24 weeks. Efficacy was reported as a favourable/unfavourable outcome and culture conversion. Safety was reported in terms of death and frequency of adverse events of special interest.

**RESULTS:**

Of the 127 articles identified, 13 were included. The proportion of favourable outcomes reported was 76−100%, and the median time to culture conversion was 4−6 weeks. Culture conversion rates ranged from 80–100% by the end of 3 months of treatment, regardless of the type of drug resistance. Treatment completion rates in the operational research studies varied between 18–93%. Safety events were not proportionate among the studies included, possibly due to the differing linezolid dosing (more frequent in the 1,200 mg dose regimen).

**CONCLUSION:**

Our review supports the use of Pa-based regimens in patients with DR-TB. The results indicate that Pa-based regimens are efficacious with tolerable safety profile in DR-TB patients.

## Graphical Abstract

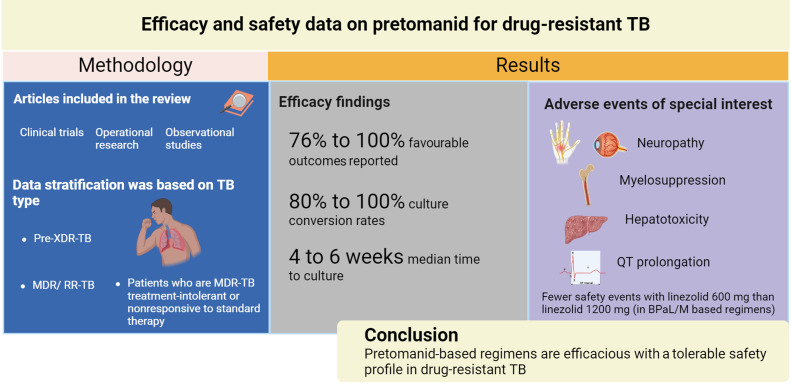

TB remains a significant global concern, with nearly 1.3 million deaths in 2022.^1^ Over 80% of deaths occur in low- and middle-income countries, with the highest burden observed in sub-Saharan Africa, Asia, Central and Eastern Europe.^2^ Patients with weakened immune systems, diabetes, undernutrition and addiction (to alcohol or tobacco) have the highest risk of contracting TB.^3^ The emergence of drug-resistant TB (DR-TB) has become an escalating concern in recent decades. This is due to inappropriate or ineffective use of antimicrobials without drug susceptibility testing (DST), inadequate adoption of systematic treatment approaches for both drug-susceptible TB (DS-TB) and DR-TB, the emergence of HIV in regions with pre-existing DR-TB, poor adherence to treatment, limited availability of effective drugs, and the transmission of drug-resistant strains.^4,5^ In December 2022, WHO published updated consolidated treatment guidelines on DR-TB,^6^ including a revision of the definition of extensively drug-resistant TB (XDR-TB) and the introduction of a new category of pre-XDR-TB. Pre-XDR-TB is defined as TB caused by *Mycobacterium tuberculosis* (MTB) strains that meet the definition of multidrug-resistant (MDR) or rifampicin-resistant TB (RR-TB) and are additionally resistant to any fluoroquinolone (FQ) antibiotic. This category highlights the seriousness of DR-TB and the need for effective management and treatment strategies.^7^ According to WHO estimates, there are more than half a million new cases of RR- and MDR-TB reported annually,^8,9^ posing challenges for TB management. Resources required to treat the disease remain scarce,^10,11^ necessitating the need for new, shorter and oral treatment regimens^12,13^ to potentially improve treatment adherence and tolerability.

In 2019, pretomanid (Pa) was approved by the US Food and Drug Administration (US FDA) in combination with bedaquiline (BDQ) and linezolid (LZD) for the treatment of adults with pulmonary TB that is resistant to isoniazid (INH), rifampicin (RIF), an FQ, and a second-line injectable antibacterial drug, or those resistant to INH and RIF, who are treatment-intolerant or non-responsive to standard therapy. The recommended dose was 200 mg/day for 26 weeks.^14^ It was subsequently approved by the European Medicines Agency (EMA; Amsterdam, The Netherlands), India Health Authority (The Central Drugs Standard Control Organization [CDSCO]; New Delhi, India), and many other health authorities across the world.^15,16^ In December 2022, WHO updated the guidelines and recommended Pa-based regimens for the treatment of RR-/MDR-/pre-XDR-TB.^17^ In 2023, India became the first country to receive regulatory approval for Pa-based regimens for RR-TB and MDR-TB.^16^

In this review, we aim to summarise efficacy and safety data for Pa-based regimens in patients with DR-TB.

## METHODOLOGY

### Study design

A combination of structured and unstructured searches was implemented to identify relevant studies about the efficacy and safety of Pa-based regimens in DR-TB patients. An extensive literature search was conducted using the following steps: 1) developing a detailed search strategy, 2) screening and selecting studies, 3) data extraction, 4) tabular summarisation of the efficacy outcomes, and 5) tabular summarisation of safety outcomes. As this research does not qualify as a clinical study, Ethics Committee approval were not required, and no participants were involved in this study, so informed consent was not needed.

### Search strategy

Electronic databases (such as PubMed and Cochrane Library) were used to identify studies reporting on the efficacy and safety data for Pa-based regimens in DR-TB patients up to February 2024. Medical subject headings (MeSH) terms and relevant keywords, such as ‘pretomanid’, ‘2-nitro-6-(4-(trifluoromethoxy)benzyloxy)-6,7-dihydro-5H-imidazo(2,1-b)(1,3)oxazine)’, ‘PA 824’, ‘PA824 cpd’, ‘drug-resistant tuberculosis’, ‘multidrug-resistant tuberculosis’, and ‘extensively drug-resistant tuberculosis’ were used. The detailed search strategy and search strings are presented in Supplementary Data S1. An unstructured search of Google Scholar, and presentations at conferences related to Pa-based regimens was performed to fill in data gaps. Quantitative data reporting the efficacy and safety of Pa in patients with RR-, MDR-, and pre-XDR-TB were included. The efficacy variable was reported as a favourable outcome, unfavourable outcome, and culture conversion. A favourable outcome was defined as the resolution of clinical symptoms, a negative culture at the end of therapy (either 24 weeks or 26 weeks), and not being classified as an unfavourable outcome. An unfavourable outcome included death, treatment failure, treatment discontinuation, loss to follow-up, or recurrence of TB at 72 weeks after randomisation. Safety data were reported as death and frequency of adverse events (AEs) of special interest, such as peripheral neuropathy, optic neuritis, myelosuppression, hepatotoxicity and QT prolongation.

### Eligibility criteria

Of the studies identified, only clinical trials, operational research, and observational studies reporting the efficacy and/or safety of Pa-based regimens in DR-TB patients were included. All included studies should have had Pa as a part of the treatment regimen for at least 24 weeks. Studies with no efficacy or safety data about Pa, reviews, case reports, consensus statements, study protocols, and duplicate studies were excluded.

### Data extraction

The titles and abstracts of all the articles obtained from the literature search were screened and reviewed independently by ARB and SH. Duplicate articles, consensus statements, case reports and review articles were removed. In the next step, the remaining articles were retrieved for their full text and assessed using the eligibility criteria (conference abstracts were also identified and screened for eligibility). In case of disagreement during review, consensus was reached after thorough discussion. In the final step, key information such as population characteristics, the study intervention, number of patients exposed, efficacy and safety data were extracted and summarised.

## RESULTS

The database search resulted in 127 articles (PubMed, *n* = 124; Cochrane Library, *n* = 3). Of the 127 articles (structured and unstructured search), 121 were excluded as they did not meet inclusion criteria. Data from conferences (abstracts, *n* = 8) were also analysed. After reviewing the full-text articles and abstracts for eligibility, 13 studies were included for the final analysis, including three clinical studies, eight operational research (abstracts) and two observational studies. Details of data extraction are given in the Figure.

**Figure.**
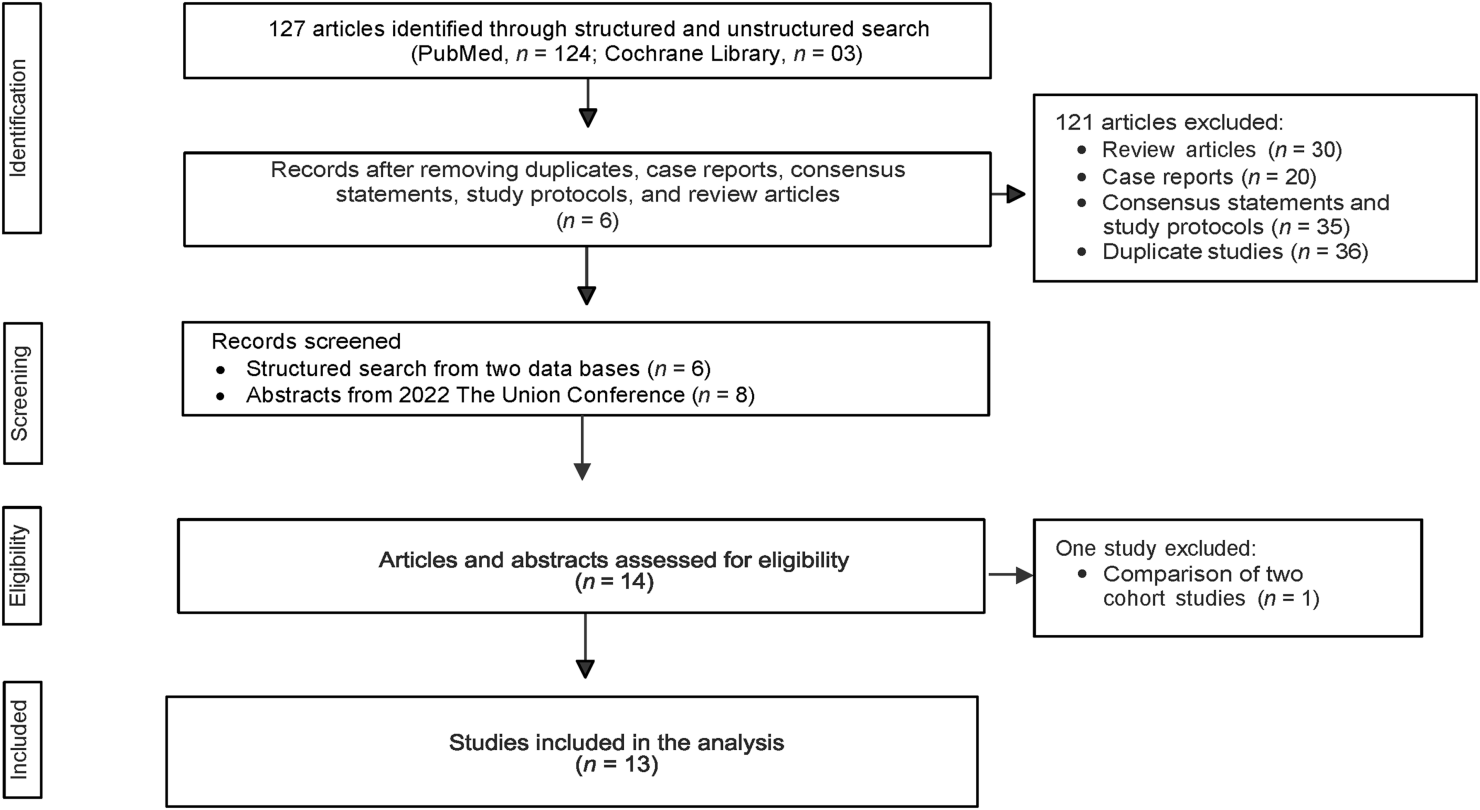
Flowchart describing the inclusion of efficacy and safety studies related to pretomanid in the final analysis.

### Study characteristics

The studies were divided into three types: clinical study, operational research and observational study (depending on the study’s nature). Data from clinical studies were further divided into patients with pre-XDR-TB, patients who were MDR-TB treatment-intolerant or non-responsive to standard therapy and patients with MDR/RR-TB based on the updated WHO TB definitions. The Pa dose was constant across all studies, i.e., 200 mg daily (Table 1).

**Table 1.** Study characteristics in the treatment of DR-TB.

First author, year, trial	Population characteristic (per study criteria)	Intervention	Patient exposed *n*
Pre-XDR-TB patients			
Conradie, 2020, Nix-TB Trial^18^	XDR-TB (resistance to INH, RIF, FQ, and an injectable)	BPaL regimen*	71
Conradie, 2022, ZeNix-TB Trial^19^	XDR-TB (resistant to RIF, an FQ, and an aminoglycoside); pre-XDR-TB (resistant to RIF and to either an FQ or an aminoglycoside)	BPaL regimen^†^	XDR-TB: 75; Pre-XDR-TB: 85
MDR-TB treatment intolerant/non-responsive to standard therapy patients			
Conradie, 2020, Nix-TB Trial^18^	MDR-TB that did not respond to treatment or for which treatment was stopped because of side effects	BPaL regimen*	MDR treatment-intolerant: 19; Non-responsive to standard therapy: 19
Conradie, 2022, ZeNix-TB Trial^19^	RR-TB that was not responsive to treatment or for which a second-line regimen had been discontinued because of side effects	BPaL regimen^†^	RR-TB, non-responsive to standard therapy: 12; RR-TB, treatment-intolerant: 9
MDR/RR-TB patients			
Nyang’wa, 2024, TB Practecal Trial^20^	RR-TB (patients were included irrespective of FQ resistance)	BPaL regimen,^‡^ BPaLM regimen, BPaLC regimen	RR-TB: 32, RR-TB + FQ-resistant TB: 80
Operational research^§¶^			
Iskandarov, 2022, Tajikistan^21^	DR-TB, including XDR-TB	BPaL regimen*	46
Lytvynenko, 2022, Ukraine^22^	Pre-XDR-TB or failure/intolerance of MDR-TB treatment	BPaL regimen*	97
Medvedieva, 2023, Ukraine^23^	Pre-XDR-TB or failure/intolerance of MDR-TB treatment	BPaL regimen*	358
Padmapriyadarsini, 2023, India^24^	Pre-XDR-TB or failure/intolerance of MDR-TB treatment	BPaL regimen^#^	125
Cervas, 2023, Philippines^45^	Pre-XDR-TB or failure/intolerance of MDR-TB treatment	BPaL regimen*	103
Mirtskhulava, 2023, Indonesia, Kyrgyzstan, the Philippines, Uzbekistan, and Vietnam^25^	Pre-XDR-TB or failure/intolerance of MDR-TB treatment	BPaL regimen*	319
Kiria, 2023, Georgia^26^	Pre-XDR-TB	BPaL regimen*	29
Waheed, 2023, Pakistan^27^	MDR/RR-TB, pre-XDR-TB	BPaL and BPaLM^‡^	263 patients
Observational study			
Goswami, 2022, CDC^29^	XDR-TB (resistance to isoniazid, rifamycin, FQ, and an injectable) MDR-TB that did not respond to treatment or for which treatment was stopped because of side effects	BPaL regimen*	Total: 20
			MDR-TB: 8;
			Pre-XDR-TB: 10;
			XDR-TB: 1;
			DS-TB: 1
Haley, 2023, BPaL Implementation Group Study^28^	Patients with RR-TB or RIF-intolerant TB	BPaL regimen*	70

* Nix-TB Trial, Iskandarov et al. (2022), Lytvynenko et al. (2022), Haley et al. (2023), Cervas et al. (2023), Mirtskhulava et al. (2023), Kiria et al. (2023): BPaL regimen, comprising BDQ 400 mg once daily for 2 weeks, followed by 200 mg three times a week for 24 weeks + Pa 200 mg daily for 26 weeks + LZD 1,200 mg daily for up to 26 weeks (dose adjustment depending on toxic effects).

† ZeNix-TB Trial: BPaL regimen, BDQ 200 mg daily for 8 weeks, then 100 mg daily for 18 weeks + Pa 200 mg daily for 26 weeks + LZD at a dose of 1,200 mg for 26 weeks or 9 weeks or 600 mg for 26 weeks or 9 weeks.

‡ TB Practecal Trial, Waheed et al (2023): BDQ dose of 400 mg daily for 2 weeks, followed by 200 mg three times per week for 22 weeks + Pa at a dose of 200 mg daily for 24 weeks + LZD at a dose of 600 mg daily for 16 weeks, followed by 300 mg daily for 8 weeks; BPaLM regimen, BPaL + moxifloxacin at a dose of 400 mg daily for 24 weeks; BPaLC regimen, BPaL + clofazimine at a dose of 100 mg daily or 50 mg if the patient weighed <30 kg for 24 weeks.

# BDQ and Pa with daily LZD 600 mg for 26 weeks (Arm 1) OR LZD 600 mg for 9 weeks, followed by 300 mg for 17 weeks (Arm 2) OR LZD 600 mg for 13 weeks, followed by 300 mg for 13 weeks (Arm 3).

§ Pre-XDR-TB under operation research: Resistance that fulfils the definition of MDR/RR-TB and that is resistant to any FQ.

¶ MDR-TB under operation research: Resistance to at least INH and RIF.

DR-TB = drug-resistant TB; XDR-TB = extensively DR-TB; INH = isoniazid; RIF = rifampicin; FQ = fluoroquinolone; MDR-TB = multidrug-resistant TB; RR-TB = rifampicin-resistant TB; B, BDQ = bedaquiline; Pa = pretomanid; L, LZD = linezolid.

Note: Mirtskhulava et al (2023), Kiria et al (2023), Goswami et al (2022): Initially used BPaL regimen,* however the dose of LZD was later reduced to 600 mg per day.

Two regimens of BDQ were used: 1) alternate day regimen (400 mg once daily for 2 weeks, followed by 200 mg thrice a week for 24 weeks); 2) daily regimen (200 mg daily for 8 weeks, then 100 mg daily for 18 weeks). The LZD dosing protocol was different in all the studies, with a maximum dose of 1,200 mg per day in the Nix study and a tolerable dose based on patient tolerability.

### Efficacy results

The efficacy of Pa-based regimens was evaluated in terms of favourable/unfavourable outcomes and culture conversion (Table 2). In pre-XDR-TB patients and patients who were MDR-TB treatment-intolerant or non-responsive to standard therapy, the proportion of favourable outcomes ranged from 77–95%,^18,19^ and 67–100%,^18,19^ respectively. The median time to culture conversion was 4−6 weeks.^18,19^ In patients with MDR/RR-TB, the proportion of favourable outcomes was between 76–00%, with 89% culture conversion observed at the end of 12 weeks.^20^ One case of recurrence (by 108 weeks of follow-up) was seen in patients with MDR/RR-TB who received bedaquiline (B), pretomanid (Pa), linezolid (L), and moxifloxacin (BPaLM), whereas recurrence with bedaquiline (B), pretomanid (Pa), linezolid (L), and clofazimine (BPaLC) regimen and bedaquiline (B), pretomanid (Pa), and linezolid (L) (BPaL) was seen in 5 and 4 patients respectively. There was only one case of treatment failure with the BPaLC regimen.^20^

**Table 2.** Efficacy details of the shortlisted articles.^*^

First author, year, name of trial	Efficacy outcome	
	Favourable/unfavourable outcome	Culture conversion
Pre-XDR-TB		
Conradie, 2020, Nix-TB Trial^18^	Favourable outcome ITT population: 63/71 (89%)	50% (at the end of 6 weeks).
Conradie, 2022, ZeNix-TB Trial^19^	Favourable outcome for XDR-TB: Overall study mITT: 63/74 (85%) Dose wise: • 1,200 mg for 26 weeks: 19/21 (90.5%) • 600 mg for 26 weeks: 18/19 (94.7%) Favourable outcome for pre-XDR-TB: Overall study mITT: 79/83 (95%) Dose wise: • 1,200 mg for 26 weeks: 17/18 (94.4%) • 600 mg for 26 weeks: 20/22 (90.9%)	Median time to culture conversion as per dose: 1,200 mg for 26 weeks: 4 weeks (IQR 2–8) 600 mg for 26 weeks: 6 weeks (IQR 3–8)
MDR-TB treatment intolerant/non-responsive to standard therapy patients		
Conradie, 2020, Nix-TB Trial^18^	Favourable outcome ITT population: 35/38 (92%)	50% (at the end of 6 weeks).
Conradie, 2022, ZeNix-TB Trial^19^	Favourable outcome for RR-TB (RR/AE-intolerant): Overall study mITT: 17/21 (81%) Dose wise: • 1,200 mg for 26 weeks: 5/5 (100%) • 600 mg for 26 weeks: 3/4 (75%)	Median time to culture conversion as per dose: 1,200 mg for 26 weeks: 4 weeks (IQR 2–8) 600 mg for 26 weeks: 6 weeks (IQR 3–8)
MDR/RR-TB		
Nyang’wa, 2024, TB Practecal Trial^20^	Unfavourable outcome BPaLM patient group: mITT population, 16/138 (12%) BPaLC patient group: mITT population, 27/115 (23%) BPaL patient group: mITT population, 15/111 (14%)	In the mITT, 107/120 patients (89%) in the BPaLM group had culture conversion at 12 weeks
Operational research		
Iskandarov, 2022, Tajikistan^21^	NA	32/33 (97%) patients showed culture conversion within 4 months
Lytvynenko, 2022, Ukraine^22^	Cure rates and treatment completion: 90/97 (93%)	NA
Medvedieva, 2023, Ukraine^23^	Cure rates and treatment completion: 65/358 (18%), ongoing treatment: 276/358 (77%), death: 5/358 (1.4%), failure: 4/358 (1.1%), loss to follow-up: 5/358 (1.4%), remaining 3 withdrawn due to baseline resistance	196/245 (80%) reached culture conversion in 3 months of treatment
Padmapriyadarsini, 2023, India^24^	Treatment completion: 125/400 (33%) in 26 weeks of treatment	112/118 (95%) culture converted by 26 weeks (40/40 in Arm 1, 36/39 in Arm 2, 36/39 in Arm 3)^†^
Cervas, 2023, Philippines^45^	Treatment completion: 58/103 (56%) finished 6 months of treatment (linezolid 1,200 mg/d; Success rate with BPaL: 97% (56)	NA
Mirtskhulava, 2023, Indonesia, Kyrgyzstan, the Philippines, Uzbekistan, and Viet Nam^25^	Cure rates and treatment completion: 138/146 (94.5%)	158/176 individuals (89.8%) reported no growth after 1 month of BPaL treatment
Kiria, 2023, Georgia^26^	Cure rates and treatment completion: 17/20 (85%).	Culture conversion: 20/20 (100%)
Waheed, 2023, Pakistan^27^	Treatment completion: 17/24 (71%) patients completed the treatment successfully	Culture conversion at 1 month: 70%, BPaL; 82% BPaLM Culture conversion at 2 months: 87%, BPaL; 92% BPaLM Culture conversion at 3 months: 91%, BPaL; 100% BPaLM
Observational study		
Goswami, 2022, CDC^29^	Completed treatment at follow-up 12 months: 19/20 (95%) with no treatment failures, recurrences, or deaths	NA
Haley, 2023, BPaL Implementation Group Study^28^	Favorable outcome: 6 months: 55/68 (80.9%)	Median time to culture conversion: 37 days (range 1–90)

* BPaL, comprising BDQ 400 mg daily for 2 weeks, followed by 200 mg three times per week for 22 weeks + Pa at a dose of 200 mg daily for 24 weeks LZD 600 mg daily for 16 weeks, followed by 300 mg daily for 8 weeks; BPaLM regimen, comprising BPaL + moxifloxacin 400 mg daily for 24 weeks; BPaLC regimen, comprising BPaL + clofazimine 100 mg daily or 50 mg if the patient weighed <30 kg for 24 weeks.

† Arm 1: BDQ and Pa with daily LZD 600 mg for 26 weeks; Arm 2: LZD 600 mg for 9 weeks, followed by 300 mg for 17 weeks; Arm 3: LZD 600 mg for 13 weeks, followed by 300 mg for 13 weeks.

XDR-TB = extensively drug-resistant TB; ITT = intent-to-treat; mITT = modified ITT; IQR = interquartile range; RR-TB = rifampicin-resistant TB; AE = adverse event; NA = not available; B, BDQ = bedaquiline; Pa = pretomanid; L, LZD = linezolid.

The efficacy outcome for operational research was evaluated based on culture conversion in patients. Operational research in Tajikistan indicated a culture conversion rate of 97% within 4 months of treatment.^21^ According to research conducted in Ukraine, 93% of patients were cured and completed their treatment.^22^ Another study in Ukraine showed 80% culture conversion rate in patients with pre-XDR-TB who completed 3 months of treatment. ^23^ A study from India reported a culture conversion rate of 95% at the end of 26 weeks of treatment.^24^ In an Asian multicounty operational research study, 90% of patients reported no growth in MGIT culture after 1 month of BPaL treatment. ^25^ A Georgian study reported a 100% culture conversion rate in a median time of 36 days. ^26^ Operational research in Pakistan showed culture conversion rates of 91% and 100% after 3 months of BPaL and BPaLM treatment, respectively, ^27^ and treatment completion rates were between 18–93%. ^27^

The proportion of favourable outcomes reported in an observational study by Haley et al. was indicated by the percentage of patients free from TB post-treatment: 81% of the patients were relapse-free in 6 months post-treatment follow-up. The median time to culture conversion was 37 days (range 1–90); 81% of patients had completed their 6-month treatment course.^28^ In another observational study from the United States by the Centers for Disease Control and Prevention (CDC), at 12 months follow-up after the initiation of BPaL treatment, 19 patients (95%) had completed treatment for TB with no treatment failures, recurrences or deaths.^29^

### Safety results

Safety data on the Pa-based regimens were described according to the organs involved.

*Peripheral neuropathy*: In the Nix-TB Trial,^18^ most peripheral neuropathy cases occurred after 8 weeks of treatment and resulted in LZD interruption, reduction or discontinuation. No peripheral neuropathy-related adverse drug reaction (ADR) led to discontinuation of the entire study regimen (Table 3).

**Table 3.** Adverse events of special interest.^*^

Adverse events of special Interest	Clinical study				
	Conradie, 2020, Nix-TB Trial	Conradie, 2022, ZeNix-TB Trial	Nyang’wa, 2024, TB Practecal Trial	Haley, 2023, BPaL Implementation Group Study	Goswami, 2022, CDC
Peripheral neuropathy	88/109 (81%)	1,200 mg for 26 weeks: 17/45 (38%) 600 mg for 26 weeks: 11/45 (24%)	—	4/68 (5.9%)	6/20 (30%)
Optic neuropathy	2/109 (1.8%)	4/45 (9%)	—	NA	3/20 (15%; vision change)
Myelosuppression	52/109 (48%)	1,200 mg for 26 weeks: 10/45 (22%) 600 mg for 26 weeks: 1/45 (2%)	Anaemia: BPaLM, 5/151 (3%); BPaLC, 1/126 (1%); BPaL, 1/123 (1%) Neutropenia: BPaLM, 3/151 (2%); BPaLC, 1/126 (1%); BPaL, 1/123 (1%) Lymphocyte count decreased: BPaLM, 1/151 (1%); BPaLC, 1/126 (1%); BPaL, 1/123 (1%)	Haematologic toxicity: 3/68 (4.4%)	—
Hepatotoxicity	17/109 (15.7%)	47/181 (26%)	BPaLM: 12/151 (8%) BPaLC: 5/126 (4%) BPaL: 5/123 (4%)	—	—
QT prolongation	6/109 (5.5%)	4/181 (2.2%)	BPaLM: 2/151 (1%) BPaLC: 2/126 (2%) BPaL: 0 patient	—	—

* BPaL, comprising BDQ 400 mg daily for 2 weeks, followed by 200 mg three times per week for 22 weeks + Pa at a dose of 200 mg daily for 24 weeks + LZD 600 mg daily for 16 weeks, followed by 300 mg daily for 8 weeks; BPaLM regimen, comprising BPaL + moxifloxacin 400 mg daily for 24 weeks; BPaLC regimen, comprising BPaL + clofazimine 100 mg daily or 50 mg if the patient weighed <30 kg for 24 weeks.

CDC = Centers for Disease Control and Prevention; NA = not available; B, BDQ = bedaquiline; Pa = pretomanid; L, LZD = linezolid.

*Optic neuropathy*: Two patients in the Nix-TB Trial^18^ and four patients in the ZeNix Trial^19^ developed optic neuropathy; in both trials, the event was resolved by the interruption or dose reduction of LZD. No episodes of optic neuropathy were observed in the TB Practecal study (Table 3).

*Myelosuppression*: In the Nix-TB Trial,^18^ 37% of patients experienced anaemia, which can be attributed to LZD. The majority of cytopenias began after 2 weeks of treatment. Overall, three patients had cytopenia that was considered serious. All three serious adverse events (SAEs) resulted in the interruption of LZD or the entire regimen, and all were resolved. In the ZeNix Trial,^19^ anaemia and neutropenia were the only two AEs with >5% incidence. Leukopenia and anaemia were observed in >10% of patients in the TB Practecal study,^20^ whereas Grade ≥3 SAE of anaemia, neutropenia and decreased lymphocyte count was seen in less than 5% of the study population (Table 3).

*Hepatotoxicity*: In the Nix-TB Trial,^18^ 21% of patients experienced ADR of higher transaminases. All of these patients were able to continue or resume therapy after interruption and complete the full course of treatment, except one patient who died due to pneumonia and sepsis (Table 3).

*ECG QT interval prolongation*: In the Nix Trial,^18^ 5.5% experienced QT prolongation. No subject was reported to have a treatment emergent QTcF exceeding 480 ms. One subject was reported to have a change from the baseline of QTcF exceeding 60 ms. QTcF prolongation for more than 500 ms was seen in respectively one and two patients from the BPaLM and BPaLC groups in the TB Practecal trial.^20^ AEs of special interest from all enrolled studies are available in Table 3. No safety details were presented for the operational research available.

*Death:* Six deaths were reported in the Nix Trial,^18^ one in the ZeNix Trial,^19^ and 13 in the TB Practecal Trial.^20^ Two deaths in the Nix Trial^18^ were considered possibly related to the study treatment, while the deaths reported in ZeNix^19^ and TB Practecal Trials^20^ were considered unrelated.

## DISCUSSION

TB is one of the leading causes of death from a single infectious disease.^30^ In 2022, an estimated 1.3 million deaths worldwide were due to TB. Although a net reduction of 8.7% in the incidence rate was observed from 2015 to 2022, the End TB Strategy is not on track.^2^ One important factor that might increase the number of DR-TB cases is the lack of tests for resistance, affecting the overall treatment of DR-TB.^31^ Only about 1 in 3 people with DR-TB receive effective treatment, which includes 20 pills per day, including injectables.^32,33^ Also, a favourable treatment outcome was observed only in 66% of patients.^34^ Misuse of antimicrobial drugs, ineffective formulations and early termination of treatment can lead to drug resistance. Limited resources and evolving profiles of DR-TB complicate the treatment. Therefore, effective management is vital to control DR-TB.^1^ Pa is a new anti-TB drug with a solid bactericidal and bacteriostatic effect. In combination with other anti-TB drugs, it is highly active against MTB.

The objective of this review was to provide a single source of all available information on the latest efficacy and safety evidence of Pa-based regimens from human studies. The article selection was aimed at providing a holistic view of the current information on the subject and included clinical trials, operational research and observational studies. All the included studies broadly fitted the DR-TB group. However, further categorisation of patients into subgroups did not perfectly align with the revised WHO definitions, as Nix^18^ and ZeNix^19^ Trials had already initiated their recruitment, whereas in the TB Practecal Trial^20^ and in eight operational research studies,^21,22^ whole-genome resistance testing was not performed. Patients from the Nix^18^ and ZeNix^19^ studies were categorised as pre-XDR-TB and MDR-TB treatment-intolerant/non-responsive to standard therapy. Although 25% of the patients from TB Practecal^20^ had FQ resistance and were potentially classified as pre-XDR-TB, we have included all the patients under the MDR/RR-TB category due to a lack of subgroup analysis for efficacy outcome. All operational research studies^21,22^ included patients from the pre-XDR-TB or MDR treatment-intolerant/non-responsive to standard therapy categories, but as the study reports are only interim reports, these were categorised separately. The Haley et al. study was an observational study in TB patients with RR-TB or RIF intolerance by the BPaL Implementation Group (BIG) for patients treated with BPaL between 14 October 2019 to 30 April 2022.^28^ The other observational study (Goswami et al.), which had minimum follow-up data of 12 months, included XDR- and MDR-TB patients treated with BPaL from August 2019 to September 2020.^29^

Efficacy outcomes were consistent across clinical studies, operational research, and observational studies. The favourable outcome rate was >85% in most DR-TB patients, irrespective of TB type. Data from a recent systematic review reported that Pa-based regimens had 46.73 times higher odds (95% confidence interval [CI] 11.76–185.70) of achieving favourable compared to unfavourable outcomes. The same author conducted another round of meta-analysis, excluding the study by Tweeds et al.^35^ to focus on the BPaL/BPaLM regimen. The results remained similar, showing higher odds (of 41.67) for achieving favourable outcomes compared to unfavourable outcomes.^36^

In most operational research studies, the number of favourable outcomes was fewer than in other studies. This may be due to a variety of reasons, including a lower number of patients who completed treatment, patients failing to meet the study criteria, extension of treatment duration by 3 months, etc. Also, all the favourable outcome calculations were based on the patient population that had completed the study treatment. The other efficacy outcome measured was culture conversion rate. The median time to culture conversion for most studies was 4−6 weeks, irrespective of TB type. Ensuring patient adherence to treatment is important because the above operational studies reported low treatment completion rates (in some cases, this could be due to the study’s ongoing nature). Therefore, there is concern about the amplification of drug resistance, especially with regimens containing a small number of drugs and short treatment duration. The dropout rates could be even higher in the real world because of greater patient support and monitoring conditions in these controlled settings.

The safety data were mainly available in three clinical studies,^18-20^ and two observational studies.^28,29^ Safety events were not comparable in all five studies. This is mainly because of the different LZD doses, ranging from 1,200 mg per day to tolerable doses and variable treatment duration of 26 weeks to 9 weeks. The disparity in the safety profiles could be attributed to several factors, such as differences in the evaluation of laboratory tests, variations in tools for detecting AEs, discrepancies in the methods used to record and classify AEs and genetic differences among the study population. Hence, a comparison of the doses of LZD from one study would help reduce these confounding factors. This issue is addressed by the ZeNix study, which investigated the efficacy and safety of varying doses of LZD (1200 mg/day and 600 mg/day) for treating DR-TB. This pivotal study builds on the findings of the earlier Nix-TB Study, which demonstrated high success rates, but also significant side effects linked to LZD. ZeNix evaluated lower doses (600 mg/day) and shorter treatment durations (9 weeks) to optimize the risk-benefit ratio. In the ZeNix study, differences in the Pa-based regimens were more apparent in LZD-associated safety measures than in efficacy measures. Treatment-emergent AEs (TEAEs) relating to peripheral neuropathy, myelosuppression and optic neuropathy were more common in the 1,200-mg, 26-week arm than in the 600-mg, 26-week; 1,200-mg, 9-week; and 600-mg, 9-week arms, indicating that TEAEs were more common at higher doses (1,200 mg) and with longer treatment durations (26 weeks). A greater percentage of participants in the 1,200 mg LZD arms required dose modifications (reduction, interruption or discontinuation) of LZD (51% and 30% in the 1,200-mg, 26-week, and 1,200-mg, 9-week arms, respectively, vs 13% in both 600-mg arms). In addition to the favourable side effect profile, the LZD 600 mg dose showed a lower incidence of bacteriological failure over 26 weeks (1 out of 45 participants) compared to 9 weeks (4 out of 45 participants). This suggests that the 600-mg, 26-week regimen had the most favourable risk-benefit profile among the four regimens studied, similar to the Nix-TB Trial, but with fewer toxic effects. To note, LZD dose and duration can be modified depending on the patient’s tolerance. Differences in safety events were more apparent, with different incidences of peripheral neuropathy, myelosuppression and LZD dose modifications. These observations are similar to findings from a recent systematic review.^36^

The Nix and the ZeNix Trials have different BDQ dosing schedules. The ZeNix study used daily dosing, supported by pharmacokinetic simulations showing comparable exposures to the Nix study’s alternative dosing. Both dosing protocols provide similar drug exposure over 6 months, with cumulative exposure being similar. Daily dosing in ZeNix is an alternative type of dosing to support adherence and facilitate treatment administration (all medicines were administered daily throughout the regimen). No direct clinical studies compare the two, but preclinical data suggest similar efficacy and safety profiles.^37^

The effect of Pa on male reproductive hormones is another area of concern. This is because Pa belongs to the nitroimidazole class of antibiotics, a chemical class that can cause male reproductive toxicity.^38^ However, safety data from four clinical trials (NC-002,^39^ NC-005,^40^ NC-006,^35^ and NC-008 [SimpliciTB; Clinical-Trials.gov Identifier: NCT03338621]; which are not part of this current review) reported no changes in male hormones, suggesting that these concerns are unlikely.

Based on the available scientific literature and comments from various experts, the CDC updated their DR-TB guidance in February 2022.^41^ The CDC recommends use of the BPaL regimen in adults with pulmonary TB that is resistant to INH, RIF, and at least one FQ (e.g., levofloxacin or moxifloxacin) or injectable (i.e., amikacin, kanamycin, capreomycin), or pulmonary TB that is resistant to INH and RIF among patients who are treatment-intolerant or non-responsive. Later in December 2022, WHO shared a rapid communication to update DR-TB treatment guidelines.^42^ The most important recommendation was the use of the 6-month BPaLM regimen instead of the 9- or 18-month regimens in MDR/RR-TB patients. In November 2023, the CDC updated the initial LZD dose in the BPaL regimen from 1,200 mg to 600 mg (based on the results of the ZeNix Trial).^43^ Recent WHO guidelines (June 2024) continue to recommend the 6-month BPaL(M) regimen for DR-TB, and discuss the use of alternative regimen and the 9-month BDQ-containing regimen.^44^ Many countries are currently working on updating local policies to include Pa-based regimens in the management of DR-TB.

### Limitations

A major limitation of this review is the absence of a meta-analysis. We did not perform a meta-analysis due to inconsistencies in patient population, study duration and endpoints (efficacy and safety) across the included studies. There was limited data available for analysis, as only 13 studies were found in the literature. The study design of these studies is not robust, as most of the studies (NiX, ZeNix, and operational research) included in the review have no clear comparator for Pa against which the observed efficacy could be assessed. No studies were available for special populations (such as pregnant, lactating or paediatric populations).

## CONCLUSION

DR-TB is an important global concern. Long treatment duration, poor adherence, poor outcomes, and adverse events worsen the situation for patients. Our study results indicate that Pa-based regimens are efficacious with tolerable safety profiles, which supports the use of newer Pa-based regimens in patients with DR-TB.

## Supplementary Material


