# Supplementary material for: Efficacy and safety data on pretomanid for drug-resistant TB
Source: IJTLD Open. 2025 Feb 1;2(2):73–82. doi: 10.5588/ijtldopen.24.0360 (PMC11827665; doi:10.5588/ijtldopen.24.0360)
Supplement: Supplementary file 1 [file ijtldopen24-0360_supplementarydata1.docx]

# Efficacy and safety data on pretomanid for drug-resistant TB

| **S.No** | **Search String** | **No of Hits** |
| --- | --- | --- |
| 1. | Pretomanid[Supplementary Concept] | 190 |
| 2. | (((Pretomanid) OR (2-nitro-6-(4-(trifluoromethoxy)benzyloxy)-6,7-dihydro-5H-imidazo(2,1-b)(1,3)oxazine)) OR (PA 824)) OR (PA824 cpd) | 400 |
| 3. | (#1) OR (#2) | 400 |
| 4. | (Extensively Drug-Resistant Tuberculosis[MeSH Terms]) OR (Tuberculosis, Multidrug-Resistant[MeSH Terms]) | 10196 |
| 5. | (((((((((((((((((((((((multidrug-resistant tuberculosis) OR (Tuberculosis, Multidrug Resistant)) OR (Tuberculosis, MDR)) OR (MDR Tuberculosis)) OR (Tuberculosis, Multi-Drug Resistant)) OR (Multi-Drug Resistant Tuberculosis)) OR (Tuberculosis, Multi Drug Resistant)) OR (Tuberculosis, Drug-Resistant)) OR (Drug-Resistant Tuberculosis)) OR (Tuberculosis, Drug Resistant)) OR (Multidrug-Resistant TB)) OR (MDR TB)) OR (Extensively drug-resistant tuberculosis)) OR (Extensively drug-resistant TB)) OR (Extensively Drug-Resistant Tuberculoses)) OR (Tuberculoses, Extensively Drug-Resistant)) OR (Tuberculosis, Extensively Drug Resistant)) OR (XDR-TB)) OR (Extremely Drug-Resistant Tuberculosis)) OR (Drug-Resistant Tuberculoses, Extremely)) OR (Drug-Resistant Tuberculosis, Extremely)) OR (Extremely Drug Resistant Tuberculosis)) OR (Extremely Drug-Resistant Tuberculoses)) OR (Tuberculoses, Extremely Drug-Resistant) | 22,389 |
| 6. | (#4) OR (#5) | 22,389 |
| 7. | (#3) AND (#6) | 229 |
| 8. | ("2021/01/01"[Date - Publication] : "3000"[Date - Publication]) | 13,466,685 |
| 9. | (#7) AND (#8) | 177 |
| 10. | #7 AND #8 Filters: Humans | 127 |
| 11. | #7 AND #8 Filters: Humans, English | 124 |
|  |  |  |
